# Extirpation of a Tumor of the Bladder

**Published:** 1875-08

**Authors:** 


### MISCELLANEOUS.

### Extirpation of a Tumour of the Bladder.

Dr. Carl Gussenbauer reports (*Boston Med. and Surg. Journal*, July 8, 1875,) the following case of myoma of the bladder which deserves attention, as the tumour was correctly diagnosticated and extirpated with an unexpectedly good result; also as the method of operation has never heretofore been employed, and, further, since microscopical examination proved the tumour to be of a variety rarely occurring in the bladder.

On June 3, 1874, D. J., a boy twelve years of age, was admitted to the clinic of Prof. Billroth, suffering, according to his father's statement, from stone in the bladder. He had been troubled for ten months. The first symptoms were pain after passing water, localized in the glans penis and in the region of the bladder. After a while severe attacks of painful micturition set in, which in the course of ten months became more frequent, and often came on so suddenly that the boy could not prevent a sudden discharge of urine. At the time of admission the patient was obliged to pass his water every ten minutes, a small quantity each time, with frequent and severe pain, partly in the region of the bladder and partly in the glans. Urine was feebly acid, slightly cloudy, but contained nothing characteristic on microscopical examination except a moderate quantity of pus corpuscles and a few cells of bladder epithelium.

On examination a tumour was noticed in the region of the bladder, to the left of the median line. It was to be felt through the abdominal walls; it was apparently about the size of the fist, was hard and somewhat sensitive on pressure, slightly movable, attached apparently to the bladder. Per rectum the tumour was also felt. On introduction of the sound it was also found to slide over an uneven surface. On careful examination it was noticed that the beak immediately on entering the bladder was pressed forward; and on attempting to move it from one side to another it always slid over an uneven tumour before reaching the back of the bladder. The combined examination with sound and finger, per rectum, proved clearly that a tumour connected with the back of the bladder hindered the movement of the sound. The consistence of the tumour was that of a fibro sarcoma, and the size that of a small fist.

The rapid growth of the tumour demanded energetic treatment, as it promised, in the state of suffering in which the patient then was, soon to end his life.

The operation of extirpation was performed on June 15. 1874, in the following way: After the patient was narcotized the lateral incision for removal of stone was made. The finger introduced into the bladder showed immediately that a tumour nearly the size of the fist, with an uneven surface, projected from the posterior wall and extended towards the top of the cavity of the bladder. Owing to its size, it was found impossible to extract the tumour, with the finger, from the perineum. A supra-pubic incision was then made, without injury to the peritoneum, and to give sufficient room both recti muscles were cut across at their insertion; also a transverse incision into the bladder was made. Prof. Billroth soon came to the conclusion, after examining with the finger, that the use of the écraseur was not practicable or desirable, as the tumour possibly might be already adherent to the peritoneum, in which case the latter would have been so injured as to delay healing. He therefore decided to tear the tumour with his finger near its base and to cut out the remainder from the wall of the bladder, after passing a ligature round to check bleeding. The extraction of the torn pieces of the tumour was not so easy, in spite of the large size of the incision, as would have been supposed. In dissecting out the pedicle it was necessary to turn the bladder partly inside out. It then appeared that the tumour took its origin from the muscular coat of the blader, but had not attacked the outer coat or the peritoneum. The plan was, in case the peritoneum had been opened, to close the hole with sutures. Two arteries were tied and the ligatures brought out through the upper incision in the badder.

The wound in the bladder was not closed, as primary intention was not probable after the tearing which the size of the tumour had made necessary. To prevent the flowing of urine over the upper wound (so often the cause of pericystitis after the supra-pubic operation), a drainage tube was drawn through the bladder and brought out at the incision in the perineum, in the expectation that the urine would flow through the tube. This proved to be correct, but only when the tube was pushed so high up that it appeared over the symphysis. The walls of the bladder were pressed together by the weight of the intestine; consequently the urine collected in the place where the resistance was the least, *i. e.*, above the bladder. If the opening in the drainage tube was at this place, the urine ran off by the perineum. If, however, the position of the tube was altered, the urine collected (as is the case always in the high operation for stone when the wound of the bladder is not closed) until it reached the level of the skin, and flowed over the abdomen, no urine at all passing through the drainage tube. I mention this apparently trival circumstance as I became convinced, on observing the course of the case, that the drainage tube especially contributed to the favorable result in the case. This was remarkably good, considering the apparently severe operation. The tripple wound caused hardly any reaction—rarely the case even in successful cases of lithomy. There was no inclination to a pericystitis or infiltration of the subcutaneous tissues, nor the slightest peritonitis. The first two days after the operation the patient's temperature was 37.8° C. (100° F.) and 38.8° C. (101.8° F.). On the third day the evening temperature rose to 39.6° C. (103.4° F.), but on the fourth day it sank to 38.2° C. (100.6° F.). On the sixth day there was no fever. On the fifth day after the operation, as the wound was granulating well, and there was no danger of infiltration of urine, the drainage tube was removed. The wound, on the twelfth day after the operation, was so small that the urine came partly by the urethra. The patient was discharged July 18, perfectly well, wearing a pad to counteract any tendency to hernia.

The tumour was eight centimeters long, four broad. Its largest circumference was eighteen centimeters, its smallest thirteen. It sat directly on the muscular layer of the bladder. Its base was seven centimeters in circumference. There was no ulceration, the surface was smooth, but an epithelial coating was not to be determined without the microscope. From its consistence, its appearance, and that of the cut surface, it would have been regarded as a soft fibroma. But the remarkable friability made it improbable that it was an ordinary fibroma. The friability was as marked as one usually sees in spindle-celled sarcomas only. But a merely superficial microscopical examination was sufficient to determine that the tumour was a myoma.—*Monthlg Abstract Med. Sciences.*

